# Dynamics of the Multiplicity of Cellular Infection in a Plant Virus

**DOI:** 10.1371/journal.ppat.1001113

**Published:** 2010-09-16

**Authors:** Serafín Gutiérrez, Michel Yvon, Gaël Thébaud, Baptiste Monsion, Yannis Michalakis, Stéphane Blanc

**Affiliations:** 1 Unité Mixte de Recherche BGPI, INRA-CIRAD-SupAgro, TA A-54/K, Campus International de Baillarguet, Montpellier, France; 2 Unité Mixte de Recherche GEMI 2724, CNRS-IRD, Avenue Agropolis, B.P. 64501, Montpellier, France; The Samuel Roberts Noble Foundation, United States of America

## Abstract

Recombination, complementation and competition profoundly influence virus evolution and epidemiology. Since viruses are intracellular parasites, the basic parameter determining the potential for such interactions is the multiplicity of cellular infection (cellular MOI), i.e. the number of viral genome units that effectively infect a cell. The cellular MOI values that prevail in host organisms have rarely been investigated, and whether they remain constant or change widely during host invasion is totally unknown. Here, we fill this experimental gap by presenting the first detailed analysis of the dynamics of the cellular MOI during colonization of a host plant by a virus. Our results reveal ample variations between different leaf levels during the course of infection, with values starting close to 2 and increasing up to 13 before decreasing to initial levels in the latest infection stages. By revealing wide dynamic changes throughout a single infection, we here illustrate the existence of complex scenarios where the opportunity for recombination, complementation and competition among viral genomes changes greatly at different infection phases and at different locations within a multi-cellular host.

## Author Summary

Viruses are fast evolving organisms for which changes in fitness and virulence are driven by interactions between genomes such as recombination, functional complementation, and competition. Viruses being intra-cellular parasites, one basic parameter determines the potential for such interactions: the cellular multiplicity of infection (cellular MOI), defined as the number of genome units actually penetrating and co-replicating within individual cells of the host. Despite its importance for virus evolution, this trait has scarcely been investigated. For example, there are only three point estimates for eukaryote-infecting viruses while the possibility that the cellular MOI may vary during the infection or across organs of a given host individual has never been conclusively addressed. By monitoring the cellular MOI in plants infected by the *Cauliflower mosaic virus* we found remarkably ample variations during the development of the infection process in successive leaf levels. Our results reveal that the opportunities for recombination, complementation and competition among viral genomes can greatly change at different infection phases and at different locations within a multi-cellular host.

## Introduction

Intracellular interactions among co-infecting viral genomes play a central role in viral evolution and ecology as they determine three important phenomena: (i) competition and selection, (ii) re-association with other genetic backgrounds through recombination, and (iii) functional complementation of (or by) other genomes. The overall intensity of these phenomena depends on the probability of encounter of the countless variants of a viral population within the multitude of individual cells composing the host. The basic parameter determining the potential for such encounters is the multiplicity of cellular infection (cellular MOI), i.e. the number of viral genomes (number of genome units) that enter and effectively replicate in individual cells. For example, a cellular MOI above 1 in a given cell corresponds to the co-infection of the same cell by several viral variants, favoring recombination, complementation, and intra-cellular competition; on the contrary, a cellular MOI of 1 will preclude these phenomena. Notably, complementation between viral genomes co-infecting individual cells has been investigated both theoretically and experimentally for the bacteriophage Φ6, and has been demonstrated to be a predominant evolutionary force which directly depends on the MOI, as defined here [1]–[5]. More generally, complementation (shared production of viral polymerase, movement proteins, suppressors of host defenses, structural proteins of the virion, etc.) is undoubtedly frequent in viral populations and is at the basis of collective actions, which largely operate at the intra-cellular level.

Empirical investigations on the cellular MOI are extremely scarce. In fact, the values for this parameter that prevail in nature remain elusive, and their putative dynamic changes during colonization of a host by a virus population have never been conclusively investigated. Formal MOI estimates have been established in only four systems: one bacteriophage [6], [7], one insect virus [8], and two plant viruses [9], [10]. For the bacteriophage and the insect virus, the MOI was considered as a single value calculated at one single time point. For plant viruses, both studies were limited to the initial onset of the host infection. Miyashita and collaborators [10] defined the number of virions infecting individual cells in a local lesion within a leaf immediately following the artificial inoculation of the virus in a single cell. González-Jara and collaborators [9] went a little further by analyzing the MOI both in the artificially inoculated leaf, as well as in the very first leaf where the virus appears through natural systemic movement. These empirical analyses provide important insights into the MOI, but at a very limited spatial and temporal scale during host invasion, thus leaving two remarkable lacunas. First, they cannot inform on whether MOI is constant and homogeneous throughout the entire host and infection process or, on the contrary, subject to ample dynamic changes in time and/or space. Such opposite situations could have totally different implications for viral population genetics (further discussed later). Second, and consequently, the estimated values might not even approximate the average MOI that could be calculated from the entire host across the whole infection process, potentially yielding a totally biased view of the reality.

The present study fills these important gaps by describing the first extensive spatio-temporal monitoring of the cellular MOI of a eukaryotic virus, the *Cauliflower mosaic virus* (CaMV), from the onset of the systemic invasion until senescence of its host plant. CaMV is an aphid-transmitted double-stranded DNA virus which replicates through reverse transcription of a genomic RNA intermediate, and is thus expected to have a high mutation rate [11], [12]. This virus has been shown to recombine extremely frequently [13], indirectly indicating an elevated cellular MOI. Our analysis at different time points and at different leaf levels demonstrates the occurrence of important dynamic changes of the MOI throughout the infection cycle, starting close to 2 early in infection, peaking at 13, and then decreasing to initial levels. Most importantly, we obtained similar MOI values under different experimental conditions of inoculum doses, plant growth, and inoculation methods - including natural inoculation by aphids - suggesting that our results are robust to experimental conditions, and thus faithfully illustrate what actually happens during a natural CaMV infection cycle.

## Results

### The method

Our aim was to evaluate the intensity with which the variants in a viral population can interact with each other at the cellular level, or, in other words, to assess how frequently these variants co-exist in individual cells. To this end, we estimated the cellular MOI and its putative dynamic changes during the invasion of turnip plants (*Brassica rapa*) by CaMV.

Host plants were co-inoculated mechanically with VIT1 and VIT3, two equi-competitive tagged CaMV variants, previously characterized in [14], [15], differing only in a 40-bp non-coding insert that allows their specific identification. In all experiments, the two variants were co-inoculated at the same time and location in order to mimic the situation where a mutant coexists with other genomes from its appearance.

The principle of the procedure in all time-course analyses was as follows (see full details in Materials and Methods). Six plants were inoculated in parallel and sampled at different time points, starting from the development of the first symptoms of systemic infection until flowering and senescence. At each sampling date a single mature leaf was sampled from the same leaf level in all plants. In each individual leaf two parameters were measured: (i) the ratio of the variants VIT1 and VIT3, and (ii) the proportion of cells infected by both variants. From these data, we derived a maximum likelihood estimate of the average number of viral genomes infecting individual cells (i.e., the MOI) at each leaf level.

In fact, assuming that the monitored viral variants infect cells at random, the probability for a given variant to enter a cell directly depends on both its frequency within the corresponding leaf and the total number of viral genomes that enter each cell (MOI). Given the known relative frequency of the variants VIT1/VIT3 within each analyzed leaf, we estimate the average MOI for which the likelihood to lead to the observed proportion of cells co-infected by the two variants is maximum. The full details and formulas for this maximum likelihood framework are given in the Materials and Methods.

### The CaMV MOI vastly changes during host colonization

Preliminary experiments were designed to define the VIT1/VIT3 ratio to be used in the inoculum in order to obtain an intermediate proportion of cells co-infected by both variants (when all cells contain both variants, it becomes impossible to estimate the MOI). The outcome of these preliminary experiments indicated a very high proportion of cells co-infected by both VIT1 and VIT3, and ample variations of this proportion at different sampling dates. Because variations were also important between repeated plants at each sampling date, these preliminary trials were principally used to adjust and better control our sampling protocol, and are thus fully described in the Materials and Methods, and shown in Figure S1 and Table S1.

In order to remove irrelevant sources of variation as much as possible, we repeated the whole time-course experiment homogenizing parameters during plant growth and leaf sampling (see Materials and Methods). In particular, the exact same leaf levels were collected in all six repeated plants, and all leaves were collected 13 days after their first appearance on the plant (when the leaves were 13 days old).

**Figure 1:**
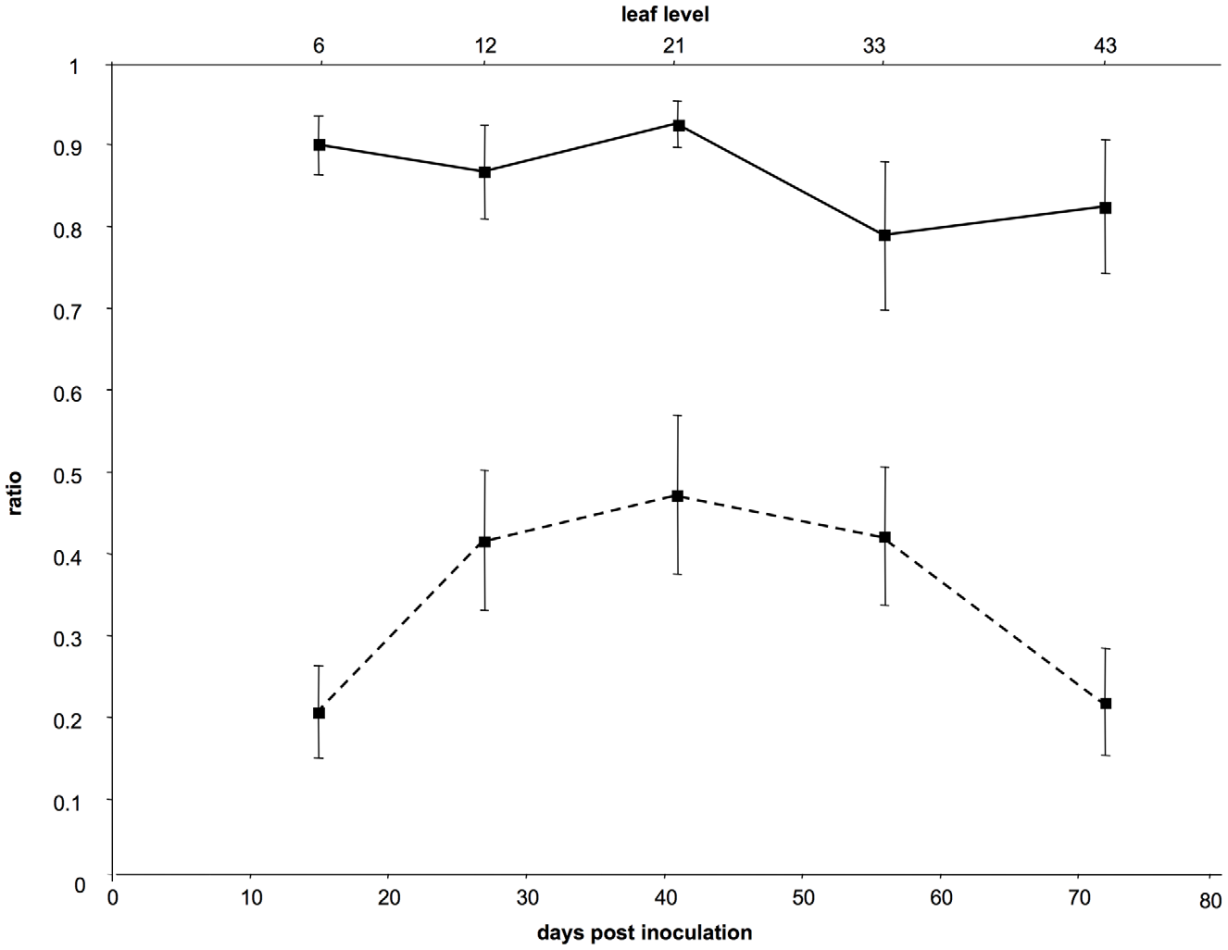
Dynamics of the frequency of VIT1 and of the proportion of cells infected by both variants. The curves on the figure represent either the average values of the frequency of VIT1 (full line) or those of the proportion of cells infected by both variants (dotted line) in different leaf levels. Days post-inoculation are indicated below and the leaf level sampled at each time point is indicated above. Bars represent standard errors. A test using a linear mixed-effects model showed no significant differences between pairs of dates for the VIT1 frequency, and the slope of the linear regression of VIT1 relative frequency versus time did not significantly differ from 0. In contrast, the same analysis showed significant differences for the proportion of cells infected by both variants (P<0.001).

**Figure 2:**
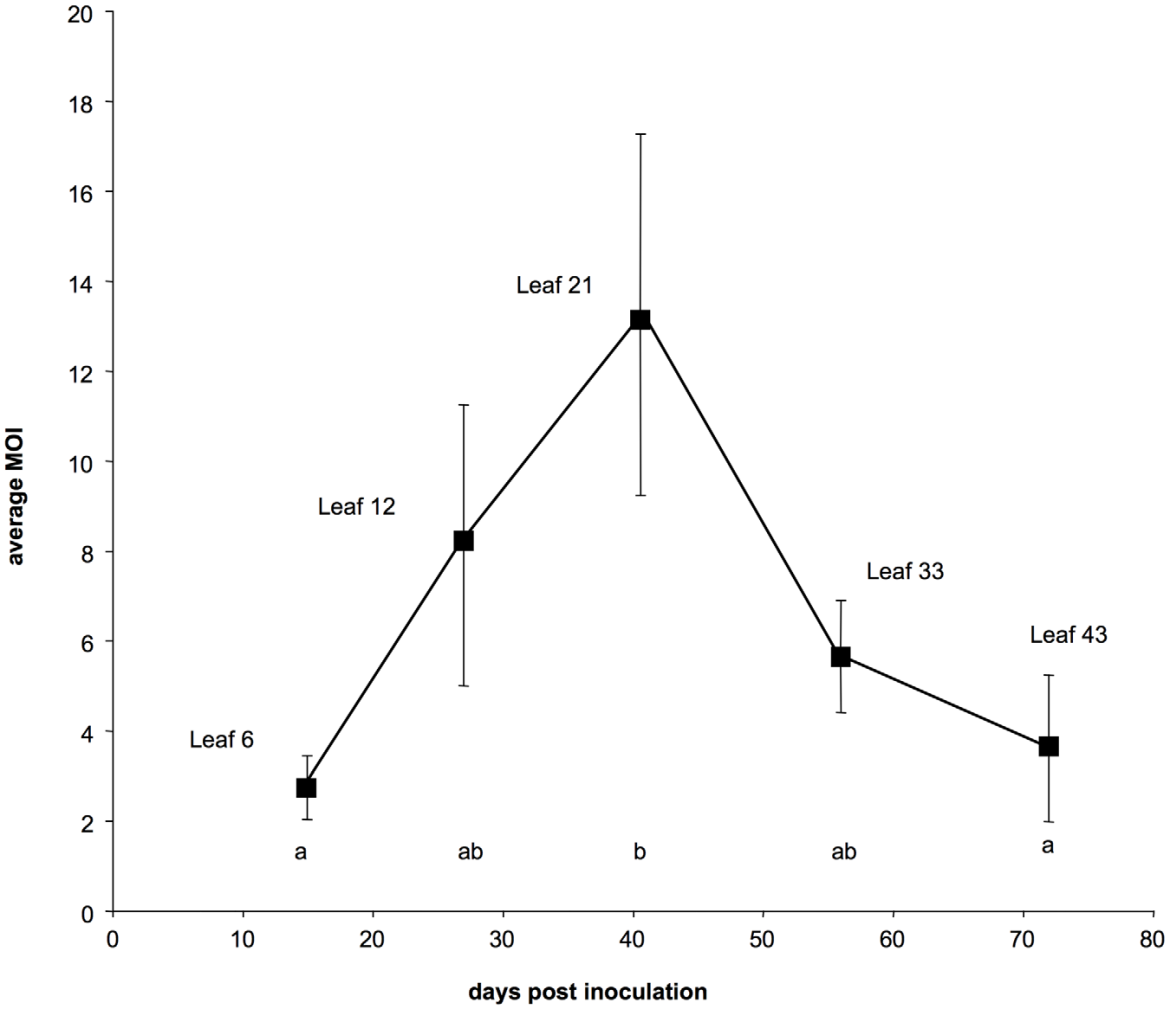
Dynamics of the multiplicity of cellular infection by *Cauliflower mosaic virus* in turnip plants. Each point represents the average estimate of the MOI over 6 infected plants at the indicated leaf level. Bars represent standard errors. Different letters between two estimates indicate significant differences (P<0.05).

The results from this controlled repetition of the time-course monitoring of CaMV cellular MOI are shown in Figures 1 and 2 (the full data set is provided in Table S2). The VIT1/VIT3 ratio within infected leaves was close to that in the initial inoculum, and remained nearly constant throughout the experiment (Figure 1 plain line). The slight differences in the VIT1 relative frequency at different time points were not statistically significant (linear mixed-effects model; *P* = 0.112; F = 2.16; dfnum = 4; dfden = 20). Moreover, the slope of the linear regression of VIT1 relative frequency versus time was not significantly different from 0 (*P* = 0.078; F = 3.46; dfnum = 1; dfden = 23), consistently with the equi-competitiveness of VIT1 and VIT3 in our experimental condition (see Materials and Methods). In contrast, the proportion of cells infected by both variants varied significantly between leaf levels (Figure 1 dotted line; linear mixed-effects model; *P* = 3.1×10^−4^; F = 8.71; dfnum = 4; dfden = 20). In line with the preliminary results presented in Figure S1, we found that the estimated MOI values (Figure 2) followed a bell-shaped curve with a peak at approximately 13 genomes per cell (in leaf level 21), and minima of around 2 at the early symptoms appearance (leaf level 6) and during flowering preceding plant senescence (leaf level 43). Variations between the six repeated plants were lower than in the preliminary experiment mentioned above, and the statistical analysis confirmed both a significant MOI increase from leaf level 6 to 21 (Tukey HSD test; P = 0.027), and a significant decrease from leaf level 21 to 43 (Tukey HSD test; P = 0.048). Because the leaves successively developing on the same plant were all analyzed at the same leaf-age, we conclude that they were infected by CaMV at a significantly different MOI.

This conclusion was further confirmed by an alternative statistical approach where the MOI in each leaf-level was estimated within a full maximum likelihood framework (described in the materials and methods) which results are presented and discussed in detail in the Supporting Online Information (Figure S2).

### The CaMV MOI is barely affected by changes in the experimental conditions

**Figure 3:**
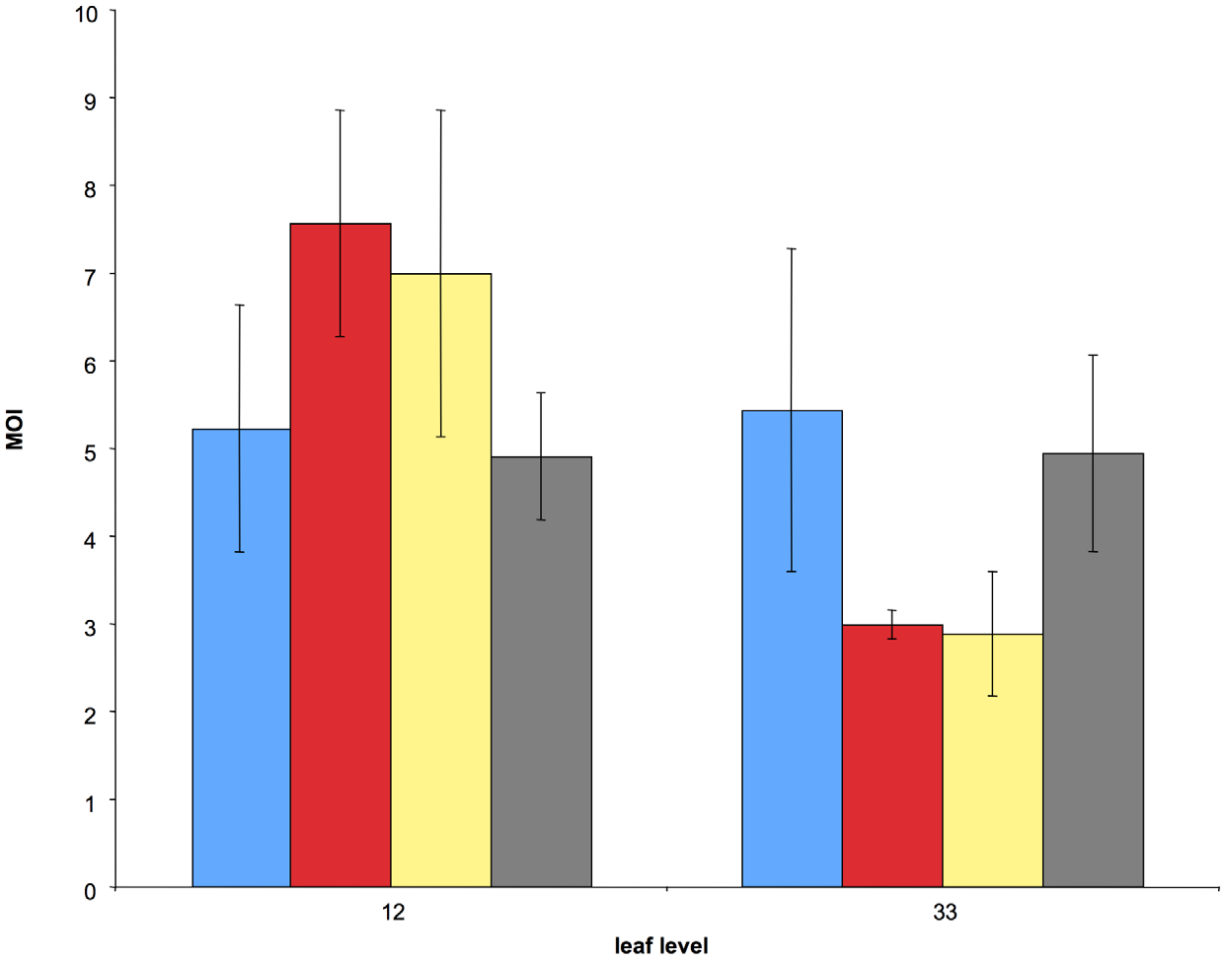
Comparison of the average cellular MOI in leaf levels 12 and 33 under four different treatments. Blue: same experimental conditions as in Fig. 2 (control); red: the inoculum dose was four times that of the control; yellow: same inoculum dose as in the control but under different growth conditions; green: same growth conditions as in the control, but plants were inoculated with aphid vectors. Bars represent standard errors.

Our next goal was to test whether our results were specific to the experimental design, in particular to the mechanical inoculation process, which is commonly used in laboratories but does not correspond to the natural mode of inoculation of CaMV. Thus, we investigated how the MOI estimates varied in different experimental conditions (Figure 3, the full dataset is provided as Supporting online Information in Table S3).

These experimental conditions included changes (i) in the plant growing conditions, (ii) in the virus dose inoculated mechanically, and (iii) in the mode of inoculation (including aphid transmission). The experimental design was similar to that in the time-course experiment described above except that, for practical reasons discussed later, only two leaf levels were sampled (leaves 12 and 33). Consequently, we could not investigate the effects of these treatments on the MOI dynamics, but we could nevertheless compare their respective values for these two leaf levels. Figure 3 shows that all conditions yielded values of the same order of magnitude as in the other experiments reported in Figure 2 (and also in Figure S1). A linear mixed-effects model (with leaf level and treatment and their interaction as fixed effects, and plant as a random effect) revealed that treatment did not affect MOI (P = 0.99; F = 0.042; dfnum = 3; dfden = 18), while leaf level did (P = 0.016; F = 7.01; dfnum = 1; dfden = 18). The interaction of leaf level and treatment was marginally significant (P = 0.0488; F = 3.19; dfnum = 3; dfden = 18). The results of the “leaf level” and “leaf level” x “treatment” interaction are driven by the two treatments shown in “red” and “yellow” in Figure 3 (increased viral dose and different growing conditions respectively). They suggest that the MOI dynamics might be shifted to the “left”, i.e. occur faster, under these conditions. While fully testing this possibility would have required more time points in all four treatments, our results strongly suggest that our estimates are robust and most likely representative of MOI values in nature. This important conclusion is particularly supported by the condition where CaMV was inoculated by aphid vectors (shown in green in Figure 3), which is the only mode of transmission reported for this virus in nature.

## Discussion

### 1-Aim of the study and appropriateness of our experimental system

We here report the first time-course analysis of the cellular multiplicity of infection of a virus invading a eukaryotic host, from the beginning to the end of the host infection process. Our experimental design, monitoring the MOI at different time points and in different locations within the host, was intended to accommodate the likely heterogeneous structure of viral populations in different organs and at different phases of the infection cycle, as suggested by previous studies both in animals [16] and plants [17]. The genetic markers used in CaMV VIT1 and VIT3 are both neutral [15] and highly stable: they are not deleted from the viral genome after at least three successive passages in host plants [14]. These properties enabled the monitoring of the MOI for a very long period (over 80 days), without biases due either to the competitive exclusion of one variant by the other, or to increasing frequency of marker-deleted genomes within the population. The flipside of the use of these markers is that the search for cells co-infected by VIT1 and VIT3 is extremely tedious and time-consuming [18], and this is precisely why we have limited the study of the robustness of our results to the experimental conditions to only two leaf levels (Figure 3). The rationale for choosing VIT1 and VIT3 markers rather than seemingly more amenable markers (such as fluorescent protein genes) allowing high throughput detection in single cells is fully explained in the Materials and Methods. We simply wish to mention here that VIT1 and VIT3 markers can be detected within infected cells for unlimited amounts of time. Upon replication, the CaMV forms characteristic and very stable electron-dense inclusion bodies (“viral factories”), where hundreds or thousands of mature viral particles accumulate and remain sequestered indefinitely [19]. In consequence, once a CaMV variant has entered a cell and replicated, it likely remains detectable by our nested-PCR procedure until cell death.

In preliminary experiments where plants were co-inoculated with both VIT1 and VIT3 at a 1∶1 ratio, we rapidly observed nearly 100% of the cells infected by both variants. This observation is extremely interesting because it indicates that the CaMV variants are not spatially segregated in contrast to most RNA plant viruses [10], [20]–[22]. Together with the equi-competitiveness of VIT1 and VIT3, this observation is consistent with the assumption that CaMV variants infect cells at random within a leaf. Thus, assuming that the number of genomes of a given viral variant entering a cell follows a Poisson distribution, which is at the basis of most statistical methods estimating the MOI [7], [8], [10], appears appropriate in the case of CaMV.

### 2-Hump-shaped evolution of cellular MOI during host infection

The cellular MOI in a given host/virus association depends *a priori* on two parameters: the number of viral infectious units available per cell (viral load), and the maximal number of these units that can effectively co-infect the same cell. Variations in these parameters should influence cellular MOI values and explain the dynamics observed in CaMV-infected plants. It is reasonable to imagine that the viral load increases over time in the plant, with a concomitant increase in the multiple infections of cells, as more and more infected leaves develop and shed virus into the phloem. However, the decline in cellular MOI late in infection contradicts this prediction. Since VIT1 and VIT3 are equi-competitive [15], this decline cannot be explained by the dominance of one variant over the other, as confirmed by the unchanged VIT1/VIT3 ratio throughout the experiment (Figure 1 and S1A). One possibility would be a host developmental or physiological effect on the MOI, related to the previously described impairment of virus infection upon flowering [23], or to the onset of a plant defense mechanism [24], [25], with a resulting drop in viral load.

Another explanation of the observed MOI pattern would be a changing balance between benefits and costs of multiple infection of cells. The benefits are basically those derived from recombination [26]–[28], and from cooperation among the genomes co-existing within the same cell (i.e. collective action and mutualistic complementation). The costs of multiple infection arise from the competition for cell resources, and from the evolution of “cheater” genotypes, better adapted to this competition than to host exploitation in single infections [1]. The best studied example of the latter phenomenon is the recurrent observation of defective interfering particles (DIPs) appearing in virus populations [29]–[32]. The CaMV recombines at very high rates in turnip [13], and cooperative behaviours in this virus exist at least during the transmission process [33]–[35] and the suppression of gene silencing [36], [37]. One could thus hypothesize that an increasing cellular MOI could benefit CaMV during the invasion of a host, up to a value (around 13) where the costs would overwhelm the benefits. For example, as indicated above, a high MOI value might increase the proportion of DIPs [7] up to a threshold were functional genomes can no longer sustain the growth of the viral population. The resulting crash of the virus load could therefore explain the MOI drop late in infection. A quantitative monitoring of the virus load within the vasculature of the plants, and an estimate of the frequency of DIPs therein, would support or disqualify these hypotheses.

### 3-Dynamic versus constant cellular MOI

The MOI values and their dynamic changes reported here cannot be directly compared with the situations in other host/virus associations, because no equivalent information is available. The MOI estimate around 4 for a baculovirus infecting lepidopteran insects possibly represents an average over the complete infection process [8]. For the sake of comparison we calculated the equivalent average MOI for CaMV by compiling the full data sets from time-course experiments shown in Figures S1 and 2, and found values of the same order of magnitude, 7.87±2.03 and 6.67±1.43 (mean±SE) respectively. Whether the value of 4 found in baculovirus-infected caterpillars resulted from a constant MOI throughout the infection cycle or represented the average of ample variations, as is the case here for CaMV, is not known. In two recent studies on plant viruses, the MOI was investigated in the artificially inoculated leaf. Very early after inoculation, the values found for the *Tobacco mosaic virus* (TMV) infecting *Nicotiana benthamiana* plants [9], and for the *Soil-born wheat mosaic virus* (SBMV) infecting *Chenopodium quinoa* plants [10] were remarkably similar (between 5 and 6). We did not analyze the inoculated leaf in our study on CaMV, because the mechanical inoculation procedure does not reflect any natural process, and how this might or might not bias the viral infection of neighboring cells is hard to evaluate. The study on TMV [9] also reported the analysis of MOI values in the first systemically infected leaf, where the virus enters via its natural route (the plant vascular system). In this leaf, the MOI of TMV was estimated to lie between 1 and 4, very close to our estimate for CaMV in leaf level 6 (mean = 2.73; SE = 1.73) which also represents the first systemically infected leaf level. Interestingly, the same authors assessed a putative time variation of the TMV MOI within this single leaf (a question not tested here on CaMV), and they concluded that the TMV MOI can change through time. However, this conclusion was challenged in the discussion by Miyashita and Kishino [10], thus leaving opened the basic question of a MOI change with time. On this important question, we here definitely demonstrate that dynamic changes of the MOI indeed occur with large amplitudes during the whole host infection by CaMV. Unfortunately, this remarkable phenomenon cannot be compared to the situation with TMV and SBMV, where the viral infection was not monitored in upper leaf-levels being systemically infected.

A dynamic MOI similar to that described here for CaMV likely occurs in other systems, as suggested in HIV by the number of proviruses per cell indicating an elevated MOI [38], and by the fluctuating rates of cell co-infection in cell cultures [39]. However, alternative scenarios are also possible since segregation and isolation of genetic variants in different cells of the same host has been repeatedly observed for several plant viruses [17], [20]–[22], [40]–[42], suggesting more stringent limits to cellular co-infection, and thus to MOI values, at least within some specific cell types, organs, or tissues.

At present, no theoretical predictions are available to fuel a discussion on the potentially different impact that a steady or a variable cellular MOI could have on the evolution of the corresponding viral populations. The few theoretical and experimental studies addressing specifically the role of MOI in the evolution of the phage Φ6 were considering low, intermediate, or high values, but always constant in a given viral line (reviewed in [5]). While we here observe ample MOI variations during host infection by CaMV, we cannot control it, and a comparison with a constant MOI is thus far impossible in this system. In contrast, other virus-host models, like phage systems, would allow the experimental evolution of lines with constant or changing MOI, with various different patterns but similar average value, and the outcome on the evolution of the average fitness in each line would be extremely interesting.

Beyond the within-host scale of virus evolution, a specific pattern of variable cellular MOI might have important implications also at a higher organization level, in a broader ecological context. For instance, in the specific case of CaMV, it is possible that populations evolve under different cellular MOI values depending on the vector species. This virus can indeed be transmitted by several aphid species [43] with different behaviors: colonizing the plant or not, feeding from lower or upper leaves, or from younger or older plants. Given the implications of the MOI for viral evolution and epidemiology, our results urgently call for a broader investigation of this important trait in a wide panel of natural virus/host associations, characterizing the values, their putative dynamic changes and the underlying mechanisms.

## Materials and Methods

### Virus and host plant

The two engineered CaMV variants, VIT1 and VIT3, have been previously characterized in detail [14]. Both are infectious full-length clones of the CaMV Cabb-S isolate [44] harboring a 40-bp DNA insert used as a specific genetic marker that can be quantified in a mixed population [14] and specifically detected within single cells [18]. Such markers were demonstrated to be stably maintained within CaMV genomes over at least three successive passages in turnip host plants [14]. Co-infecting CaMV-VIT1 and -VIT3 proved equi-competitive during turnip plant invasion [15].

The virus particles used in the inoculum were purified from plants infected with each variant individually and quantified as previously described [45]. The inoculum was prepared by mixing purified virus particles and a convenient ratio of 4/1 (VIT1/VIT3) was determined in preliminary experiments (see below). For all time-course analyses of the MOI six healthy plantlets were mechanically inoculated in parallel with 400 ng of virus particles per plantlet as previously described [18], except for conditions with a different viral dose or inoculation by aphids. When symptoms appeared on systemically infected leaves they were harvested and processed as described below.

Unless otherwise indicated turnip plants (*Brassica rapa* cv. “Just Right”) were maintained in an insect-proof growth chamber under controlled conditions (24/15°C day/night with a photoperiod of 15/9 h day/night).

### Estimation of VIT1/VIT3 ratios in individual infected leaves

The actual VIT1/VIT3 ratio in each sampled leaf was estimated from a pool of ∼3000 protoplasts per leaf, using real-time quantitative PCR (PCR conditions and primer sequences are provided in Table S4). A linear mixed-effects model, taking into account the repeated measures within each plant, was used to test for changes in VIT1 frequency between dates (fixed effect) within plants (random effect); it showed that VIT1 frequency was close to that in the mixed inoculum and varied only slightly (if at all) over time (Figure 1 and Supporting online Information Figure S1 and Table S1), confirming previous estimates of marker neutrality [15].

### Estimation of the frequency of cell co-infection by the two variants

Thirty protoplasts from each sampled leaf were analyzed individually to determine the co-occurrence of VIT1 and VIT3 genomes and thus the frequency of cell infected by both variants. The region of the CaMV genome bearing the genetic markers was amplified from each isolated cell by single-cell nested-PCR, and VIT1 and VIT3 sequences were specifically identified in the amplicons by high resolution melting analysis exactly as described previously [18]. A linear mixed-effects model, taking into account the repeated measures within each plant, was used to test if the proportion of cells infected by both variants varied between leaf-levels (fixed effect) within plants (random effect).

Despite the tediousness of the single-cell detection of such markers [18], we have altogether analyzed over 3400 individual cells (Table S1, S2 and S3). The use of another type of markers, based on the insertion of genes encoding fluorescent proteins such as GFP (green) and RFP (red) into viral genomes, would have provided a straightforward high-throughput approach to visualize their presence within single cells, using for example epifluorescence microscopy (on tissues or extracted protoplasts). However, in contrast to the VIT1 and VIT3 markers used here, such fluorescent markers have a number of drawbacks which limits their usefulness for studies such as that presented in this paper: (i) currently available fluorescent protein genes cannot be introduced in CaMV and in other viruses with an icosahedral shell, because of the limited size of the encapsidated genome [46], [47]; (ii) GFP can diffuse autonomously from cell to cell in plants [48], a phenomenon potentially misleading in identifying cells infected with a GFP-expressing virus; (iii) two *Tobacco mosaic virus* variants, respectively expressing GFP and RFP, proved differentially competitive in co-infected plants [9], and we observed a similar phenomenon with *Turnip mosaic virus* (unpublished results); (iv), these GFP or RFP markers are often rapidly deleted from the genomes of plant viruses [49], [50], a phenomenon incompatible with their monitoring throughout the infection process.

### Estimation of the MOI

The MOI was inferred with a maximum likelihood procedure from (i) the relative proportion of the two variants measured in each sampled leaf, and (ii) in the same leaf, the number of cells infected by both variants among the infected cells.

Assuming that cell infections occur in a random and independent manner for both variants, the number of genomes of a given variant entering a cell follows a Poisson distribution with a parameter equal to the product between the cellular MOI (λ) and the relative frequency of this variant in the sampled leaf (*p_i_,* for VIT1). The null class of each Poisson distribution corresponds to the probability of not being infected by the corresponding variant. Thus, in the *i* ^th^ sampled leaf, the probability for a given infected cell to be co-infected by the two variants is 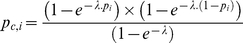, and, among the *N_i_* infected cells observed within this leaf, the number of co-infected cells has a binomial distribution with parameters *N_i_* and *p_c,i_*. The corresponding likelihood function is: 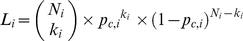, where *k_i_* is the observed number of cells infected by both variants within the *i* ^th^ sample. The MOI within each sample is then easily derived as the maximum likelihood estimate of λ. A linear mixed-effects model, taking into account the repeated measures within each plant, was used to test if the MOI varied between treatments and between dates (fixed effects), within each plant (random effect). The significance of MOI differences between specific levels of the factors was investigated using Tukey's HSD (honest significant difference) method.

The above-described statistical approach was confronted to an alternative analysis, which consisted in working within a full maximum likelihood framework providing one MOI estimate at each date from all 6 replicates. This full maximum likelihood framework is derived from the likelihood function 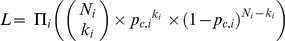, with profile-likelihood confidence intervals.

The MOI parameter (λ) was first held constant across all plants and leaf levels, and we used likelihood ratio tests to test whether allowing variation in λ across leaf levels (dates) significantly improved the likelihood of the model. We also similarly tested whether we had a plant effect, though we were much less interested by this factor which should be modeled as a random effect (as indicated above). The outcome of both analyses are shown and discussed in the Supporting Online Information (Figure S2).

All statistical procedures were implemented in the statistical software R [51].

### Time-course analyses of the MOI

As a first exploratory experiment, the plants were inoculated with a VIT1/VIT3 mixture at a 1/1 ratio and sampled twice, at early and later stages of the infection. The proportion of cells infected by both variants was around 30% in leaves collected 17 days post infection (dpi), and reached nearly 100% in upper leaves collected 60 dpi (not shown). This result interestingly suggested that cell co-infection increased with time, but that it could become frequent enough to “saturate” our experimental system when a 1/1 variant ratio was used in the inoculum: when both variants are detected in nearly all cells it becomes impossible to obtain an accurate MOI estimate with our method.

In a second time-course experiment, we thus decided to use a 4/1 ratio for VIT1 and VIT3. At 21, 42, 60 and 84 dpi, fully expanded leaves were collected near the apex of six plants infected in parallel. The results shown in Figure S1A indicate that the relative ratio of VIT1 and VIT3 was indeed close to 4/1 in infected leaves, and remained approximately constant throughout the experiment. Most interestingly, the average proportion of cells infected by both variants dramatically increased in successive sampling times but remained below saturation, suggesting both that the 4/1 ratio was appropriate and that important changes in the MOI may occur during the invasion of the host. The calculated average MOI values showed a dynamic pattern, starting at lows around 1, sharply increasing up to 13 and then decreasing late in infection (Figure S1B). Unfortunately, important variation between the six replicated plants at each sampling date resulted in too wide confidence intervals, and the statistical analysis failed to confirm the significance of the observed bell-shaped pattern (the full data set is provided in Table S1).

In order to reduce to a minimum the variations between repeated plants, we very precisely adjusted the leaf-sampling protocol during time-course experiments. The development of every new leaf was periodically scrutinized in six plants infected in parallel, to record the dates of their first appearance in the center of the rosette, and to later estimate their respective age at the sampling time. Leaves were numbered so that the first true leaf (above cotyledons) was leaf level 1. The mixture of CaMV VIT1/VIT3 purified virions (ratio 4/1) was inoculated to leaf levels 3 and 4, and the first leaf level showing systemic symptoms homogeneously distributed all over its surface was leaf level 6. The induction of flowering was generally observed around 40 dpi, when leaf 30 appeared. Senescence of individual leaves started when they were approximately 35 days old, whatever the leaf level considered. At each of five time points, one identical leaf level was sampled in the six replicated plants. Selected leaf levels corresponding to the five time points were levels 6, 12, 21, 33 and 43. All leaves were sampled at the same age (13 days after their apparition on the plant) to improve comparison among leaf levels. At this age, all cells within the leaf were likely infected as indicated by the high proportion of CaMV-positive cells found during PCR analysis of individual cells (Table S1 and Table S2 in Supporting online Information). Moreover, 13 days old leaves had already gone through the physiological sink-to-source transition that stops import of photo-assimilates and viruses from the phloem [52].

Finally, to limit interference of the sampling process with plant development and systemic infection, several evenly distributed leaf discs (0.8 cm Ø), amounting solely 20% of the total leaf surface, were collected from each leaf. Protoplasts were extracted from each sampled leaf as previously described [18], [53].

### Testing the effect of different experimental conditions on the CaMV MOI

Four treatments were compared for their putative impact on MOI values. To limit potential sources of variation, the experiments were carried out in parallel with the previous experiment on the MOI dynamics and with the same batch of plantlets and inoculum. In all treatments, 6 turnip plants were co-inoculated with VIT1 and VIT3 and the leaves were sampled when they were 28 days old. Sampling was performed exactly as described above except that, for practical reasons, only two leaf levels were sampled (leaf levels 12 and 33). We reasoned that limiting this experiement to two sampling points could provide enough resolution to address the question of a possible MOI difference in different experimental conditions. In three treatments plants were kept in the same growth chamber as for the experiment shown in Figure 1 and 2. The first treatment corresponded to a mechanical inoculation exactly as above, the second to the mechanical inoculation with a 4X dose, and the third to a more natural inoculation by aphid vectors. For the latter, 20 individuals of the aphid *Myzus persicae (Sulz.)* were fed on a plant co-infected by the two viral variants and then released on the fourth leaf of healthy plantlets as previously described [54]. Finally, in the fourth treatment plants were mechanically inoculated with a 1X dose but maintained in a greenhouse where they were exposed to approximately 16 hours sunlight and higher temperatures. Under these conditions the rate of leaf appearance was nearly identical to that in the growth chamber, but total biomass was multiplied by three and flowering started approximately one week earlier (not shown).

## Supporting Information

Figure S1 Preliminary evaluation of the dynamics of CaMV cellular MOI in turnip. A) Dynamics of the average values of both the frequency of the VIT1 variant (full line) and the frequency of cell co-infected by VIT1 and VIT3 (dotted line) at different days post-inoculation in six plants. Bars represent standard errors. B) Dynamics of the multiplicity of infection of cells (cellular MOI) in turnip plants infected by *Cauliflower mosaic virus*, derived from A as described in the text. Each point in A and B represents the average estimates in a leaf level near the apex over six infected plants. Bars represent standard errors. The corresponding full data set is presented in Table S1.
(0.19 MB TIF)

Figure S2 Comparison of two distinct statistical approaches for the estimation of the cellular MOI of CaMV during a host plant infection. The graph in A corresponds to that shown in Figure 2 where the MOI was evaluated individually in each of the 6 repeated plants and then averaged over replicates at each time point. The vertical red bars represent confidence intervals to allow straightforward comparison with B. The graph in B corresponds to the second statistical approach described in the Materials and Methods (results not shown in the manuscript), where the MOI was inferred at each time point as a single maximum likelihood estimate from the whole data set obtained from the six plants. This analysis also reveals a highly significant date (leaf-level) effect, confirming the main conclusion of our study. The vertical red bars represent profile-likelihood confidence intervals. Here, the significance of MOI differences between each pair of dates was assessed with likelihood ratio tests; for each subset of data corresponding to a given pair of dates, the likelihood with only one MOI estimate for both dates was compared to the likelihood with one MOI estimate for each date. Holm's correction for multiple comparisons was applied. Different letters between two estimates indicate significant differences (P<0.05).
(0.25 MB TIF)

Table S1 Full data set of the analysis of cell co-infection by variants VIT1 and VIT3 at four time points after inoculation, and of the VIT1 frequency at each sampling point. This data set corresponds to the analysis presented in Figure S1.
(0.62 MB DOC)

Table S2 Full data set of the analysis of cell co-infection by variants VIT1 and VIT3 in five leaf levels, and VIT1 frequency in each sampled leaf. This data set corresponds to the analysis presented in Figures 1 and 2.
(0.62 MB DOC)

Table S3 Full data set of the analysis of cell co-infection by variants VIT1 and VIT3 in two leaf levels, and of VIT1 frequency in each sampled leaf under four different experimental conditions. This data set corresponds to the analysis presented in Figure 3.
(0.60 MB DOC)

Table S4 Sequences of the primers used in the quantification of the ratio VIT1/VIT3 and PCR conditions.
(0.05 MB DOC)
