# Supplementary figures and images for: Dynamics of the Multiplicity of Cellular Infection in a Plant Virus
Source: PLoS Pathog. 2010 Sep 16;6(9):e1001113. doi: 10.1371/journal.ppat.1001113 (PMC2940754; doi:10.1371/journal.ppat.1001113)

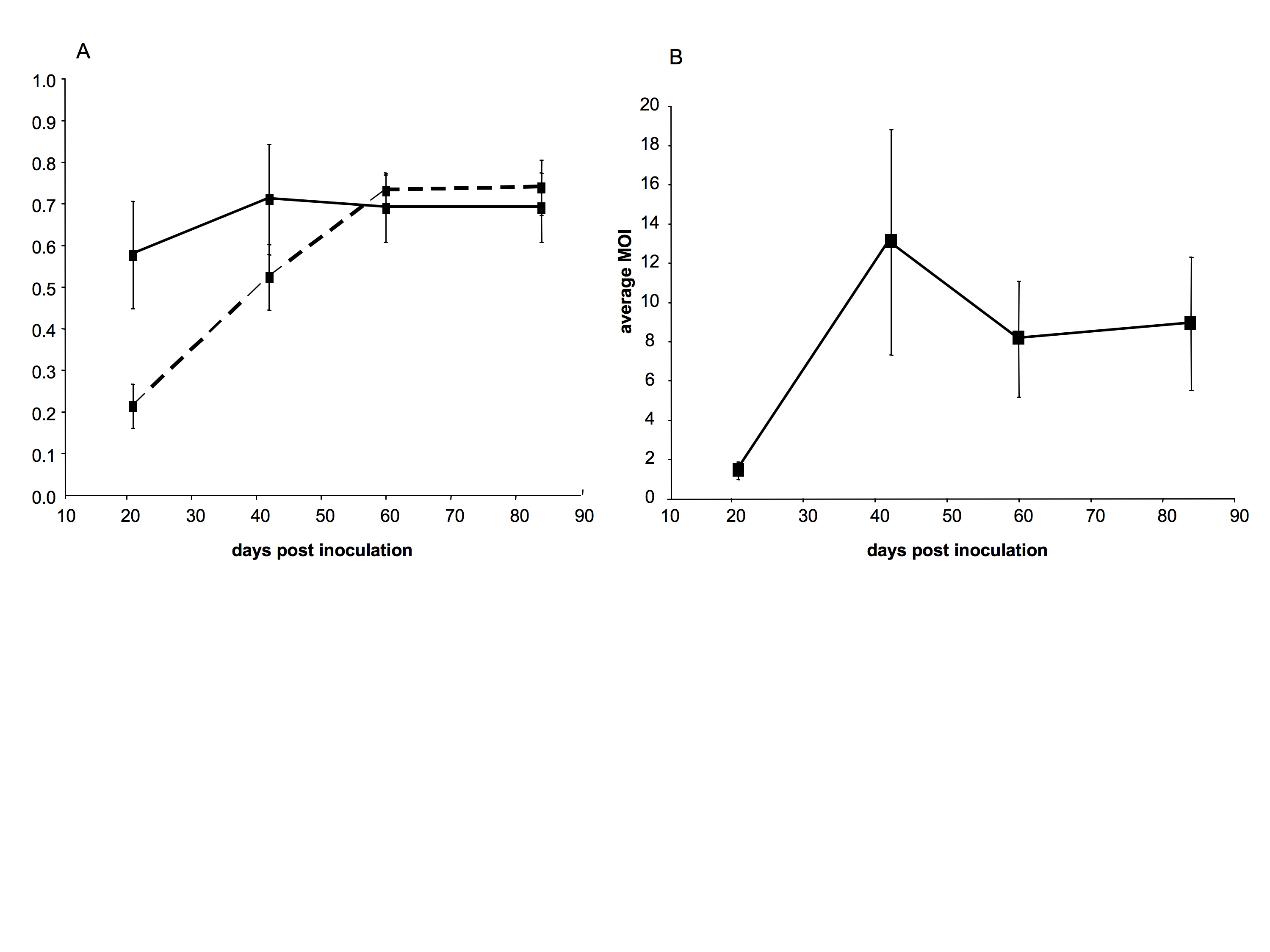

Supplement: Figure S1 — Preliminary evaluation of the dynamics of CaMV cellular MOI in turnip. A) Dynamics of the average values of both the frequency of the VIT1 variant (full line) and the frequency of cell co-infected by VIT1 and VIT3 (dotted line) at different days post-inoculation in six plants. Bars represent standard errors. B) Dynamics of the multiplicity of infection of cells (cellular MOI) in turnip plants infected by Cauliflower mosaic virus, derived from A as described in the text. Each point in A and B represents the average estimates in a leaf level near the apex over six infected plants. Bars represent standard errors. The corresponding full data set is presented in Table S1. (0.19 MB TIF) [file ppat.1001113.s001.tif]

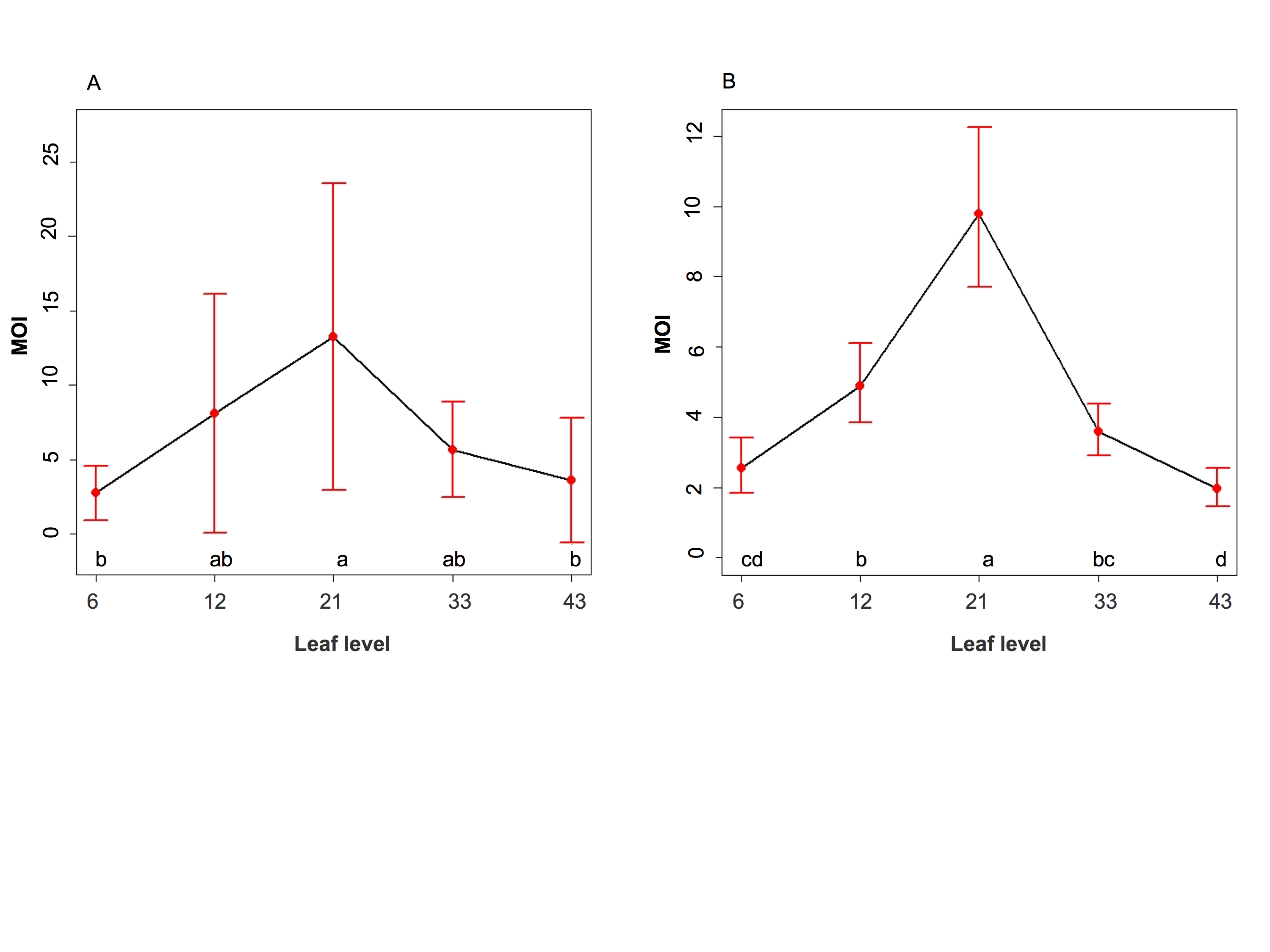

Supplement: Figure S2 — Comparison of two distinct statistical approaches for the estimation of the cellular MOI of CaMV during a host plant infection. The graph in A corresponds to that shown in Figure 2 where the MOI was evaluated individually in each of the 6 repeated plants and then averaged over replicates at each time point. The vertical red bars represent confidence intervals to allow straightforward comparison with B. The graph in B corresponds to the second statistical approach described in the Materials and Methods (results not shown in the manuscript), where the MOI was inferred at each time point as a single maximum likelihood estimate from the whole data set obtained from the six plants. This analysis also reveals a highly significant date (leaf-level) effect, confirming the main conclusion of our study. The vertical red bars represent profile-likelihood confidence intervals. Here, the significance of MOI differences between each pair of dates was assessed with likelihood ratio tests; for each subset of data corresponding to a given pair of dates, the likelihood with only one MOI estimate for both dates was compared to the likelihood with one MOI estimate for each date. Holm's correction for multiple comparisons was applied. Different letters between two estimates indicate significant differences (P<0.05). (0.25 MB TIF) [file ppat.1001113.s002.tif]
