# Supplementary material for: Dynamics of the Multiplicity of Cellular Infection in a Plant Virus
Source: PLoS Pathog. 2010 Sep 16;6(9):e1001113. doi: 10.1371/journal.ppat.1001113 (PMC2940754; doi:10.1371/journal.ppat.1001113)
Supplement: Table S1 — Full data set of the analysis of cell co-infection by variants VIT1 and VIT3 at four time points after inoculation, and of the VIT1 frequency at each sampling point. This data set corresponds to the analysis presented in Figure S1. (0.62 MB DOC) [file ppat.1001113.s003.doc]

**Table S1.** Full data set corresponding to the analysis presented in Figure S1.

| **Sampling date**  **(days post-inoculation)** | **Plant** | **Number of cells with amplified virus** | **Co-infected**  **cells** | **Absence of**  **amplification^a^** | **VIT1 frequency** |
| --- | --- | --- | --- | --- | --- |
| 21 | 1 | 25 | 4 | 5 | 0,220 |
|  | 2 | 30 | 12 | 0 | 0,150 |
|  | 3 | 30 | 1 | 0 | 0,920 |
|  | 4 | 30 | 6 | 0 | 0,790 |
|  | 5 | 31 | 5 | 0 | 0,700 |
|  | 6 | 31 | 10 | 0 | 0,680 |
| 42 | 1 | 30 | 22 | 0 | 0,600 |
|  | 2 | 25 | 17 | 5 | 0,140 |
|  | 3 | 24 | 8 | 6 | 0,990 |
|  | 4 | 30 | 20 | 0 | 0,910 |
|  | 5 | 31 | 9 | 0 | 0,970 |
|  | 6 | 30 | 13 | 0 | 0,650 |
| 60 | 1 | 27 | 23 | 3 | 0,430 |
|  | 2 | 28 | 20 | 1 | 0,460 |
|  | 3 | 30 | 25 | 0 | 0,750 |
|  | 4 | 28 | 19 | 0 | 0,950 |
|  | 5 | 29 | 20 | 2 | 0,800 |
|  | 6 | 26 | 16 | 3 | 0,750 |
| 84 | 1 | 29 | 28 | 2 | 0,430 |
|  | 2 | 26 | 21 | 4 | 0,460 |
|  | 3 | 26 | 12 | 4 | 0,750 |
|  | 4 | 25 | 18 | 6 | 0,950 |
|  | 5 | 27 | 20 | 4 | 0,800 |
|  | 6 | 30 | 22 | 1 | 0,750 |

Full data set of the analysis of cell co-infection by variants VIT1 and VIT3 at four time points after inoculation, and of the VIT1 frequency at each sampling point.

^a^The absence of VIT1 and VIT3 in a few cells could be due to the absence of infection or to occasional failure of the detection method, as previously discussed (1).
