# Supplementary material for: Dynamics of the Multiplicity of Cellular Infection in a Plant Virus
Source: PLoS Pathog. 2010 Sep 16;6(9):e1001113. doi: 10.1371/journal.ppat.1001113 (PMC2940754; doi:10.1371/journal.ppat.1001113)
Supplement: Table S2 — Full data set of the analysis of cell co-infection by variants VIT1 and VIT3 in five leaf levels, and VIT1 frequency in each sampled leaf. This data set corresponds to the analysis presented in Figures 1 and 2. (0.62 MB DOC) [file ppat.1001113.s004.doc]

**Table S2**. Full data set corresponding to the analysis presented in Figures 1 and 2.

| **Leaf level** | **Sampling date**  **(days post-inoculation)** | **Plant** | **Number of cells with amplified virus** | **Co-infected**  **cells** | **Absence of amplification^a^** | **VIT1 frequency** |
| --- | --- | --- | --- | --- | --- | --- |
| 6 | 15 | 1 | 31 | 4 | 0 | 0.912 |
|  |  | 2 | 27 | 10 | 1 | 0.725 |
|  |  | 3 | 28 | 3 | 0 | 0.927 |
|  |  | 4 | 29 | 10 | 2 | 0.915 |
|  |  | 5 | 28 | 1 | 3 | 0.971 |
|  |  | 6 | 31 | 8 | 0 | 0.939 |
| 12 | 27 | 1 | 28 | 11 | 1 | 0.845 |
|  |  | 2 | 29 | 21 | 1 | 0.614 |
|  |  | 3 | 29 | 10 | 2 | 0.961 |
|  |  | 4 | 25 | 12 | 5 | 0.823 |
|  |  | 5 | 24 | 2 | 7 | 0.979 |
|  |  | 6 | 27 | 13 | 4 | 0.971 |
| 21 | 41 | 1 | 29 | 20 | 0 | 0.948 |
|  |  | 2 | 27 | 21 | 0 | 0.797 |
|  |  | 3 | 27 | 13 | 0 | 0.976 |
|  |  | 4 | 28 | 11 | 1 | 0.892 |
|  |  | 5 | 25 | 3 | 0 | 0.965 |
|  |  | 6 | 30 | 11 | 0 | 0.967 |
| 33 | 56 | 1 | 30 | 15 | 1 | 0.492 |
|  |  | 2 | 30 | 20 | 0 | 0.531 |
|  |  | 3 | 30 | 17 | 0 | 0.817 |
|  |  | 4 | 29 | 10 | 0 | 0.959 |
|  |  | 5 | 26 | 2 | 3 | 0.985 |
|  |  | 6 | 27 | 10 | 0 | 0.943 |
| 43 | 72 | 1 | 29 | 4 | 0 | 0.665 |
|  |  | 2 | 30 | 15 | 1 | 0.493 |
|  |  | 3 | 30 | 8 | 0 | 0.878 |
|  |  | 4 | 30 | 4 | 0 | 0.954 |
|  |  | 5 | 26 | 1 | 3 | 0.973 |
|  |  | 6 | 30 | 7 | 0 | 0.977 |

Full data set of the analysis of cell co-infection by variants VIT1 and VIT3 in five leaf levels, and VIT1 frequency in each sampled leaf.

^a^The absence of VIT1 and VIT3 in a few cells could be due to the absence of infection or to occasional failure of the detection method, as previously discussed (1).
