# Supplementary material for: Dynamics of the Multiplicity of Cellular Infection in a Plant Virus
Source: PLoS Pathog. 2010 Sep 16;6(9):e1001113. doi: 10.1371/journal.ppat.1001113 (PMC2940754; doi:10.1371/journal.ppat.1001113)
Supplement: Table S3 — Full data set of the analysis of cell co-infection by variants VIT1 and VIT3 in two leaf levels, and of VIT1 frequency in each sampled leaf under four different experimental conditions. This data set corresponds to the analysis presented in Figure 3. (0.60 MB DOC) [file ppat.1001113.s005.doc]

**Table S3**. Full data set corresponding to the analysis presented in Figure 3.

| **Treatment^a^** | **Leaf level** | **Plant^b^** | **Number of cells with amplified virus** | **Co-infected**  **cells** | **Absence of amplification^c^** | **VIT1 frequency** |
| --- | --- | --- | --- | --- | --- | --- |
| 1 | 12 | 1 | 30 | 5 | 0 | 0.893 |
|  |  | 2 | 30 | 21 | 1 | 0.124 |
|  |  | 3 | 29 | 5 | 1 | 0.499 |
|  |  | 4 | 30 | 12 | 1 | 0.823 |
|  |  | 5 | 27 | 4 | 2 | 0.994 |
|  |  | 6 | 26 | 7 | 1 | 0.845 |
|  | 33 | 1 | 28 | 2 | 1 | 0.63 |
|  |  | 2 | 27 | 15 | 2 | 0.491 |
|  |  | 3 | 28 | 7 | 0 | 0.801 |
|  |  | 4 | 26 | 8 | 1 | 0.967 |
|  |  | 5 | 28 | 9 | 0 | 0.957 |
|  |  | 6 | 31 | 5 | 0 | 0.978 |
| 2 | 12 | 1 | 26 | 11 | 0 | 0.956 |
|  |  | 2 | 31 | 18 | 0 | 0.832 |
|  |  | 3 | 28 | 18 | 0 | 0.838 |
|  |  | 4 | 29 | 11 | 0 | 0.922 |
|  |  | 5 | 31 | 22 | 0 | 0.836 |
|  | 33 | 1 | 26 | 5 | 1 | 0.909 |
|  |  | 2 | 30 | 18 | 0 | 0.632 |
|  |  | 3 | 31 | 12 | 0 | 0.831 |
|  |  | 4 | 29 | 5 | 2 | 0.937 |
|  |  | 5 | 28 | 12 | 1 | 0.831 |
| 3 | 12 | 1 | 27 | 10 | 1 | 0.873 |
|  |  | 2 | 30 | 15 | 0 | 0.892 |
|  |  | 3 | 26 | 3 | 0 | 0.953 |
|  |  | 4 | 26 | 10 | 0 | 0.962 |
|  |  | 5 | 30 | 17 | 0 | 0.911 |

The table S3 continues in the next page

| **Treatment^a^** | **Leaf level** | **Plant^b^** | **Number of cells with amplified virus** | **Co-infected**  **cells** | **Absence of amplification^c^** | **VIT1 frequency** |
| --- | --- | --- | --- | --- | --- | --- |
| 3 | 33 | 1 | 29 | 7 | 0 | 0.718 |
|  |  | 2 | 28 | 7 | 0 | 0.871 |
|  |  | 3 | 26 | 3 | 0 | 0.938 |
|  |  | 4 | 30 | 15 | 1 | 0.868 |
|  |  | 5 | 31 | 7 | 0 | 0.927 |
| 4 | 12 | 1 | 29 | 9 | 0 | 0.16 |
|  |  | 2 | 30 | 3 | 0 | 0.979 |
|  |  | 3 | 29 | 9 | 0 | 0.897 |
|  |  | 4 | 30 | 26 | 1 | 0.264 |
|  |  | 5 | 31 | 26 | 0 | 0.385 |
|  |  | 6 | 30 | 6 | 0 | 0.042 |
|  | 33 | 1 | 27 | 5 | 1 | 0.199 |
|  |  | 2 | 30 | 17 | 0 | 0.861 |
|  |  | 3 | 30 | 17 | 0 | 0.911 |
|  |  | 4 | 29 | 23 | 0 | 0.62 |
|  |  | 5 | 29 | 20 | 0 | 0.511 |
|  |  | 6 | 31 | 7 | 0 | 0.052 |

Full data set of the analysis of cell co-infection by variants VIT1 and VIT3 in two leaf levels, and of VIT1 frequency in each sampled leaf under four different experimental conditions.

^a^ The four treatments are described in the main text

^b^ One plant died in the experimental conditions 2 and 3.

^c^ The absence of VIT1 and VIT3 in a few cells could be due to the absence of infection or to occasional failure of the detection method, as previously discussed (1).
