# Supplementary material for: Dynamics of the Multiplicity of Cellular Infection in a Plant Virus
Source: PLoS Pathog. 2010 Sep 16;6(9):e1001113. doi: 10.1371/journal.ppat.1001113 (PMC2940754; doi:10.1371/journal.ppat.1001113)
Supplement: Table S4 — Sequences of the primers used in the quantification of the ratio VIT1/VIT3 and PCR conditions. (0.05 MB DOC) [file ppat.1001113.s006.doc]

**Table S4**: Sequences of the primers used in the quantification of the ratio VIT1/VIT3. Approximately 3000 protoplasts were used as matrix in a PCR reaction amplifying a fragment encompassing the marker region. The conditions and primers used in this first PCR are described in detail in (1). Five microlitres of this first PCR (as described in (1)) were then used as matrix in a second real-time PCR reactions, targeting specific sequences of either VIT1 or VIT3 genomes, and using the primers shown in the Table S4. Real-time PCR was performed using an Mx3005P Real-Time PCR System (Stratagene) in the presence of SYBR-green. The real-time PCR conditions were standard (SYBR-Green I core reagent protocol) and all reagents were provided in the SYBR-Green I core reagent kit (Stratagene).

| Variant | Primers | |
| --- | --- | --- |
|  | Forward | Reverse |
| VIT1 | 5’-TAACAAGGTCCTGCCCCTTA | 5’-ACTCCGAAGGGTCTTTGCTT |
| VIT3 | 5’-TAGCCCAAAACAAAGCCTTATTAG | 5’-AATGCTCATACTAGACCAGAACC |

Reference cited:

1. Yvon M, Monsion B, Martin JP, Gutiérrez S, & Blanc S (2009) PCR-based amplification and analysis of specific viral sequences from individual plant cells. Journal of Virological Methods doi:10.1016/j.jviromet.2009.04.016
